# The Association between Maternal Smoking during Pregnancy and Childhood Obesity Persists to the Age of 9–10 Years

**DOI:** 10.2188/jea.JE20081012

**Published:** 2009-05-05

**Authors:** Kohta Suzuki, Daisuke Ando, Miri Sato, Taichiro Tanaka, Naoki Kondo, Zentaro Yamagata

**Affiliations:** 1 Department of Health Sciences, School of Medicine, University of Yamanashi, Chuo, Yamanashi, Japan; 2 Department of Physical Education, National Defense Academy, Yokosuka, Kanagawa, Japan

**Keywords:** smoking, pregnancy, life styles, obesity, fetal programming

## Abstract

**Background:**

We previously reported that a number of factors related to maternal lifestyle during early pregnancy, including smoking, are associated with childhood obesity at 5 years of age. In the present study, we investigated whether the association with maternal smoking persisted to the age of 9–10 years.

**Methods:**

The study population comprised children born between April 1, 1991 and March 31, 1999, and their mothers. The dependent variables—childhood overweight and obesity at 5 and 9–10 years of age—were defined according to internationally acknowledged cut-off values. Maternal smoking during early pregnancy was used as the independent variable.

**Results:**

Mothers who completed a specifically designed questionnaire gave birth to a total of 1644 infants during the study period. Anthropometric data were collected from 1302 of these children during medical checkups at 9–10 years of age (follow-up rate: 79.2%). Maternal smoking during early pregnancy was associated with obesity in 9- to 10-year-old children (adjusted odds ratio, 1.91; 95% confidence interval, 1.03–3.53). However, the point estimates at the age of 9–10 years were considerably lower than those at the age of 5 years.

**Conclusions:**

Our results suggest that fetal environment, including exposure to maternal smoking, continues to be associated with childhood obesity at the age of 9–10 years.

## INTRODUCTION

The increasing prevalence of childhood obesity will lead to marked increases in the number of overweight adults and higher incidences of obesity-related diseases, including coronary heart disease, high blood pressure, and adult-onset type 2 diabetes.^1^ Many studies have identified risk factors for childhood obesity, including eating behavior and physical activity.^2^ In addition, the findings of some studies on the fetal programming of chronic diseases, including obesity-related diseases, are consistent with the Barker hypothesis, which states that fetal adaptations to intrauterine undernutrition may have permanent and specific short- and long-term effects on the development of various organ systems, including the cardiovascular and metabolic systems.^3^^–^^6^ However, no study on fetal programming has examined the effects of the factors influencing intrauterine undernutrition, such as maternal smoking during pregnancy, maternal body mass index, and weight gain during pregnancy. Several investigators have suggested that maternal smoking during pregnancy increases the child’s risk of obesity, during childhood and/or adulthood.^7^^–^^13^ We previously reported that maternal smoking during pregnancy may influence the onset of obesity and overweight in 5-year-old children in Japan.^14^ We also suggested that children whose mothers skipped breakfast before pregnancy were more likely to become obese and overweight^14^ because skipping breakfast may lead to inadequate nutritional intake.^15^ Because maternal smoking during pregnancy and skipping breakfast may both be associated with intrauterine undernutrition and fetal programming, these maternal habits are important factors for consideration in investigations of childhood obesity and overweight. Although there have been many longitudinal studies on these associations,^16^ no study has compared results obtained at 2 different time points from the same cohort.

In this study, we used data obtained from a prospective cohort survey to investigate whether the associations of maternal smoking and skipping breakfast during pregnancy with childhood obesity persist to the age of 9–10 years.

## METHODS

### Participants and study design

The study population comprised children who were born between April 1, 1991 and March 31, 1999 in Koshu City, Yamanashi Prefecture, Japan, and their mothers. All are participants in Project Koshu (formerly Project Enzan), an ongoing prospective cohort study of pregnant women and their children in rural Japan, which commenced in 1988. Details of this project were described in our previous report.^14^ First, we conducted a questionnaire-based survey to investigate the lifestyles of the expectant mothers who visited the city office to register their pregnancy. Informed consent was obtained from all participants before the survey. Next, we administered a questionnaire regarding the lifestyle habits of these mothers and their children at each medical checkup of the children. During the checkups, we also obtained data on the growth and physical characteristics of the children.

This study was approved by the Ethical Review Board of Yamanashi University School of Medicine, and was conducted in accordance with the “Guidelines Concerning Epidemiological Research” (Ministry of Education, Culture, Sports, Science and Technology and the Ministry of Health, Labour and Welfare, Japan), with the cooperation of the Koshu City administration office.

### Collection of data on exposures

Data on maternal lifestyle immediately before pregnancy and smoking status during early pregnancy were obtained from the mothers by administering a self-report questionnaire at the time of pregnancy registration. In the study region, over 80% of expectant mothers registered their pregnancy during the first trimester, and almost all expectant mothers registered by the 18th gestational week. We recorded maternal lifestyle habits at the point “immediately” before pregnancy because almost all pregnant women experience nausea and vomiting during early pregnancy,^17^^,^^18^ which may cause them to change their usual activities.^17^ In this study, we used the following items derived from the findings of our previous study as independent variables^14^: maternal age and body mass index (BMI); sleep duration (<8 or ≥8 h/d); smoking status during early pregnancy (smoker, former smoker, or never smoker); and breakfast habits (eating breakfast daily or not). Other lifestyle factors were not included in this study as independent variables because they were not significantly associated with childhood obesity and overweight in our previous study.^14^ The body height and weight of women at the time of pregnancy registration were measured and recorded in the Maternal and Child Health Handbook by an obstetrician or a midwife. We used BMI as a parameter for the evaluation of maternal obesity. The maternal BMI was calculated, according to World Health Organization (WHO) standards, as body weight (kg)/height (m^2^).

### Outcome

Data on the height and body weight of the children were obtained from physical measurements collected during their medical checkup at 5 years of age. These parameters were measured again during medical checkups for grade 4 children at elementary schools, ie, when the children were aged 9–10 years. Height was measured using a stadiometer (unit: 0.1 cm), and body weight was measured using conventional weighing scales (unit: 100 g).

Obesity and overweight in children aged 5 years and 9–10 years are generally defined on the basis of BMI.^10^^,^^19^^,^^20^ The definitions of childhood obesity and overweight have been established previously^21^ and are based on international data obtained from 6 large, nationally representative cross-sectional surveys on growth from Brazil, Great Britain, Hong Kong, the Netherlands, Singapore, and the United States. BMIs of 25 and 30 are widely accepted as the adult cut-off points for overweight and obesity, respectively.^21^

### Statistical analysis

We first used the chi-square test to assess the association of maternal smoking during early pregnancy and other lifestyle habits prevalent immediately before pregnancy with childhood obesity and overweight. Although we previously observed an association at 5 years of age in the same population,^14^ not all the participants could be followed up at 9–10 years of age. Therefore, we used a new dataset to evaluate this association at 5 years of age and to examine the continuity of this association from 5 years of age to 9–10 years of age.

We subsequently used multiple logistic regression analysis to adjust the variables and the confounding factors, ie, maternal age and BMI. These analyses were based on the procedures described in our previous study.^14^ All analyses were conducted using SAS software, version 9.1 (SAS Institute Inc., Cary, North Carolina, USA).

## RESULTS

### Participants

Mothers who completed the questionnaire gave birth to a total of 1644 babies during the study period. Anthropometric data were collected from 1239 of these children during medical checkups when they were 5 years old (follow-up rate: 75.4%). Among these children, 37 (3.0%) were obese and 135 (10.9%) were overweight. Among 61 (4.9%) of these children, there was a history of maternal smoking during early pregnancy.

Anthropometric data were also collected from 1302 of these children during medical checkups at 9–10 years of age (follow-up rate: 79.2%). Among these children, 58 (4.5%) were obese and 217 (16.7%) were overweight. Among 71 (5.5%) of these children, there was a history of maternal smoking during early pregnancy.

We compared the characteristics of smoking and nonsmoking mothers and their children (Table 1). The birth weight of the children whose mothers had smoked during early pregnancy was significantly lower. Moreover, these children were significantly more likely to be small-for-gestational-age infants.

**Table 1.** Comparison of characteristics of smoking and nonsmoking mothers

Variables	Smoking mothers	Nonsmoking mothers	*P* value^*^
Maternal age (years)	27.8 ± 4.6	28.9 ± 4.2	0.02
Registration of pregnancy (weeks)	12.8 ± 5.4	10.7 ± 3.3	<0.001
Maternal body mass index (kg/m^2^)	21.0 ± 3.6	20.8 ± 2.8	0.58
Birth weight of infant (g)	2898 ± 471	3069 ± 411	<0.001
Gestational age at birth (weeks)	38.8 ± 1.4	39.0 ± 1.4	0.30

(Means ± Standard Deviation)			

Sex of infant			
​ Male	53	761	0.38
​ Female	44	761	

Intrauterine growth			
​ Small-for-gestational-age	16	100	<0.001
​ Appropriate- or Large-for-gestational-age	81	1416	

*P values for continuous variables were calculated by using the *t* test; *P* values for categorical variables were calculated by using the chi-square test.

### Crude relationship between maternal lifestyle and childhood overweight and obesity at 5 years of age (Tables 2 and 3)

The crude odds ratios (ORs) and 95% confidence intervals (CIs) for maternal lifestyle habits during early pregnancy that were associated with the weight status of the children at age 5 years are listed in Tables 2 (obesity) and 3 (overweight).

**Table 2.** Adjusted odds ratio (OR) and 95% confidence interval (CI) for maternal lifestyle factors that affected childhood obesity at age 5 years

Lifestyle factor	n*	Number of obese children	Number of non-obese children	Crude		Adjusted^†^	
	
				OR	95% CI	OR	95% CI
Smoking during early pregnancy	1218			6.09	(2.65–13.99)	5.04	(1.82–13.92)
​ Current smoker		8	53				
​ Former smoker or Never smoker		28	1129				

Sleep duration	1239			0.36	(0.16–0.79)	0.45	(0.18–1.08)
​ More than 8 h/d		8	523				
​ Less than 8 h/d		29	679				

Breakfast consumption	1223			2.41	(1.21–4.80)	3.55	(1.52–8.25)
​ Occasionally skip		13	218				
​ Never skip		24	968				

*n, number of participants who answered this question.

^†^Adjusted by maternal age, maternal body mass index, smoking status, sleep duration, and breakfast consumption.

**Table 3.** Adjusted odds ratio (OR) and 95% confidence interval (CI) for maternal lifestyle factors that affected childhood overweight at age 5 years

Lifestyle factor	n*	Number of overweight children	Number of normal weight children	Crude		Adjusted^†^	
	
				OR	95% CI	OR	95% CI
Smoking during early pregnancy	1218			2.84	(1.54–5.25)	2.82	(1.41–5.64)
​ Current smoker		15	46				
​ Former smoker or Never smoker		119	1038				

Sleep duration	1239			0.59	(0.40–0.86)	0.69	(0.45–1.05)
​ More than 8 h/d		43	488				
​ Less than 8 h/d		92	616				

Breakfast consumption	1223			1.76	(1.17–2.65)	1.99	(1.23–3.20)
​ Occasionally skip		37	194				
​ Never skip		97	895				

*n, number of participants who answered this question.

^†^Adjusted by maternal age, maternal body mass index, smoking status, sleep duration, and breakfast consumption.

The prevalence of childhood obesity was significantly higher among children with a history of maternal smoking during early pregnancy than among those without such a history (crude OR, 6.09; 95% CI, 2.65–13.99). The prevalence of obesity was also significantly higher among children whose mothers did not eat breakfast daily during early pregnancy than among those whose mothers had eaten breakfast daily (crude OR, 2.40; 95% CI, 1.21–4.80). The results obtained for the association between childhood overweight and maternal lifestyle were similar to those of the obesity analysis. However, the point estimates for childhood overweight were lower than those for childhood obesity.

### Adjusted relationship between maternal lifestyle and childhood overweight and obesity at 5 years of age (Tables 2 and 3)

We next conducted a multiple logistic regression analysis to adjust for confounding factors, ie, maternal BMI and maternal age, and to further analyze the significance of the associations between maternal lifestyle factors (smoking status, sleep duration, and breakfast consumption during early pregnancy) and childhood obesity (Table 2) and overweight (Table 3). The analysis revealed that maternal smoking was associated with overweight in the children at the age of 5 years (adjusted OR, 2.82; 95% CI, 1.41–5.64). Children whose mothers had skipped daily breakfast were more likely to become overweight (adjusted OR, 1.99; 95% CI, 1.23–3.20). Furthermore, children whose mothers had smoked during early pregnancy exhibited an independent elevated risk for obesity when compared with children whose mothers were former or never smokers (adjusted OR, 5.04; 95% CI, 1.82–13.92). Similarly, children whose mothers did not eat breakfast daily during early pregnancy were more likely to become obese (adjusted OR, 3.55; 95% CI, 1.52–8.25).

### Crude relationship between maternal lifestyle and childhood overweight and obesity at 9–10 years of age (Tables 4 and 5)

The crude odds ratios (ORs) and 95% confidence intervals (CIs) for maternal lifestyle habits during early pregnancy that affected the weight status of the children at age 9–10 years are listed in Tables 4 (obesity) and 5 (overweight).

**Table 4.** Adjusted odds ratio (OR) and 95% confidence interval (CI) for maternal lifestyle factors that affected childhood obesity at age 9–10 years

Lifestyle factor	n*	Number of obese children	Number of non-obese children	Crude		Adjusted^†^	
	
				OR	95% CI	OR	95% CI
Smoking during early pregnancy	1282			4.06	(1.96–8.42)	2.56	(1.02–6.38)
​ Current smoker		10	61				
​ Former smoker or Never smoker		47	1164				

Sleep duration	1302			0.76	(0.44–1.32)	1.05	(0.57–1.95)
​ More than 8 h/d		21	530				
​ Less than 8 h/d		37	714				

Breakfast consumption	1287			1.94	(1.09–3.45)	1.99	(1.01–3.94)
​ Occasionally skip		18	231				
​ Never skip		40	998				

*n, number of participants who answered this question.

^†^Adjusted by maternal age, maternal body mass index, smoking status, sleep duration, and breakfast consumption.

**Table 5.** Adjusted odds ratio (OR) and 95% confidence interval (CI) for maternal lifestyle factors that affected childhood overweight at age 9–10 years

Lifestyle factor	n*	Number of overweight children	Number of normal weight children	Crude		Adjusted	
	
				OR	95% CI	OR	95% CI
Smoking during early pregnancy	1282			2.22	(1.30–3.77)	1.91	(1.03–3.53)
​ Current smoker		21	50				
​ Former smoker or Never smoker		193	1018				

Sleep duration	1302			0.69	(0.51–0.94)	0.76	(0.54–1.06)
​ More than 8 h/d		76	475				
​ Less than 8 h/d		141	610				

Breakfast consumption	1287			2.03	(1.46–2.83)	2.15	(1.47–3.16)
​ Occasionally skip		64	185				
​ Never skip		151	887				

*n, number of participants who answered this question.

^†^Adjusted by maternal age, maternal body mass index, smoking status, sleep duration, and breakfast consumption.

The prevalence of childhood obesity was significantly higher among the children whose mothers had smoked during early pregnancy than among those whose mothers had not smoked (crude OR, 4.06; 95% CI, 1.96–8.42). It was also significantly higher among children whose mothers did not eat breakfast daily during early pregnancy than among children whose mothers had not skipped breakfast (crude OR, 1.94; 95% CI, 1.09–3.45). The results regarding the association between childhood overweight and maternal lifestyle factors were similar to those of the obesity analysis. However, the point estimates for childhood overweight were lower than those for childhood obesity.

### Adjusted relationship between maternal lifestyle and childhood overweight and obesity at 9–10 years of age (Tables 4 and 5)

In this analysis, maternal smoking status was associated with overweight in the children aged 9–10 years (adjusted OR, 1.91; 95% CI, 1.03–3.53). In addition, the children whose mothers did not eat breakfast daily were likely to become overweight (adjusted OR, 2.15; 95% CI, 1.47–3.16). Furthermore, the children whose mothers had smoked during early pregnancy exhibited an independent elevated risk for obesity when compared with children whose mothers were former or never smokers (adjusted OR, 2.56; 95% CI, 1.02–6.38). Similarly, the children whose mothers did not eat breakfast daily during early pregnancy were more likely to become obese (adjusted OR, 1.99; 95% CI, 1.01–3.94).

## DISCUSSION

In this prospective cohort study of Japanese participants, we analyzed data collected from pregnant mothers at the time of pregnancy registration (ie, when the children were fetuses) and followed the children to age 9–10 years. The main finding of this study was that the association of maternal smoking during pregnancy with childhood obesity at 5 years of age persisted to 9–10 years of age. These results are consistent with those of a study by Montgomery and Ekbom,^7^ which assessed the impact of maternal smoking during pregnancy on adult obesity (the National Child Development Study [NCDS] cohort). Although our point estimates were considerably higher than those obtained in previous studies, the difference in the effect sizes might be due to differences between studies with respect to the participants’ ages. In addition, the results of the present study are similar to and consistent with those of our previous study.^14^

Childhood obesity can result from childhood lifestyle factors, such as dietary habits and physical activity,^2^ and the effects of these environmental factors on childhood obesity may be more pronounced at the age of 9–10 years than at the age of 5 years. Therefore, the effects of conditions present during the fetal stage or infancy on childhood obesity may no longer be present at the age of 9–10 years. Indeed, our point estimates for the association between maternal smoking during pregnancy and childhood obesity at the age of 9–10 years were considerably lower than those obtained at the age of 5 years.

It has been postulated that smoking affects childhood obesity via intrauterine exposure to smoke, which could result in the birth of an undernourished newborn baby. This nutritional deprivation may lead to increased nutrient absorption later and, ultimately, postnatal obesity. It has been reported that undernutrition during pregnancy increases the risk of adult obesity,^22^ causes intrauterine growth retardation, and increases the risk of abnormal glucose tolerance.^23^ Our results were consistent with these reports.

It is believed that there is a positive dose–response association between both the duration and quantity of maternal smoking and childhood obesity.^24^ It is important to note, therefore, that not every woman who smoked during early pregnancy continued to smoke up to delivery in this study.^24^ In our study, women who smoked during early pregnancy comprised women who quit smoking during early pregnancy, those who quit smoking during late pregnancy, and those who continued to smoke up to delivery. Therefore, our results also suggest that maternal smoking cessation during pregnancy affects childhood obesity and overweight.

Our results suggest that a mother’s not eating breakfast daily immediately before pregnancy is an independent risk factor for childhood obesity and overweight. Williams suggested that nutrient intake was substantially more likely to be inadequate in people who skipped breakfast than in those who did not.^15^ Therefore, women who do not eat breakfast daily may be undernourished. Remacle et al demonstrated that adulthood obesity principally occurs in people who are malnourished during early gestation,^22^ and our results are consistent with theirs. Although many studies have been conducted to clarify the relation between undernutrition in pregnant mothers and the development of obesity in their children, most of these studies fail to consider that low birth weight and intrauterine growth retardation may be caused by maternal smoking during pregnancy.^25^^,^^26^ Therefore, the association between low birth weight and childhood obesity may be confounded by maternal smoking. In this regard, our results are extremely valuable for clarifying the mechanism involved in the fetal programming of obesity-related diseases.

We believe that our study has certain strengths. Many birth cohort studies have been conducted worldwide; however, most of these have not investigated the effects of maternal lifestyle habits such as smoking during early pregnancy. Moreover, almost all pregnant women in Japan register their pregnancy at a city office in order to obtain health care services during pregnancy. Consequently, we were able to obtain data from almost every pregnant woman in the study region during the study period because we collected data at the time of pregnancy registration (ie, during the fetal stage of the child’s life). We consider this to be one of the main advantages of our study.

Furthermore, because the children whose mothers had completed the questionnaire during early pregnancy were followed until the age of 5 and then to the age of 9–10 years, the follow-up period for a participant was approximately 10 years, and our total study period was approximately 18 years. Although it is usually quite difficult to follow study participants over such an extended period, the follow-up rate of our study was relatively high (79.2% at 9–10 years of age). This high rate can be attributed to the fact that most of the city’s population had not migrated elsewhere and that we were able obtain data on the height and body weight of the children at age 9–10 years from physical measurements taken during medical checkups for grade 4 children, which are conducted in all the elementary schools in Koshu city. This is another advantage of our study.

Finally, by using data from different periods of childhood growth, we were able to confirm that the association between maternal lifestyle habits during early pregnancy and childhood obesity is observable from the time the children are 5 years old and continues to be so until they are at least 9–10 years old. We believe that our results, which confirm the association between maternal lifestyle habits during pregnancy and childhood obesity, are thus very important.

This study does, however, possess certain limitations. In order to examine the continuity of the association between maternal smoking during pregnancy and childhood obesity, we could have examined continuity on the basis of monitoring individual participants rather than a study population. If we had used the former method, however, we would have been unable to maintain such a high follow-up rate and to externally validate our results, because the follow-up rate at 5 years of age was lower than that at 9–10 years of age. Therefore, we preferred to use all collected data and to examine continuity in the study population as a whole.

Although we designed a questionnaire to obtain data on maternal lifestyle habits such as smoking during early pregnancy, the validity of this questionnaire was not examined. However, a previous study demonstrated that pregnant women reported their own smoking habits very accurately.^27^ On the basis of this report, we believe that our results are valid. Another limitation of this study is that we did not obtain data on the height and weight of the fathers of the children in this study. Therefore, the effects of paternal genetic factors could not be investigated. Although we lacked data on paternal BMI, parental weight status—which reflects the genetic predisposition of the children to obesity and overweight—was partially addressed by the inclusion of maternal BMI in the analysis. We were also unable to obtain data on complications arising in the women, weight gain during pregnancy, and fetal abnormalities because this study was not conducted in a clinical setting. With regard to the analyses, we were unable to adjust for the socioeconomic status of the participants, which might be a potential confounding factor. However, we believe that the effect of this was relatively small because Japanese socioeconomic differentials are not as large as those in many foreign countries, including the United States.^28^ Finally, we could not obtain data on maternal smoking trends at various stages of pregnancy. Therefore, we could not examine the possibility of a dose–response association between maternal smoking during pregnancy and subsequent childhood obesity in this study.

In conclusion, our results suggest that both the smoking status and dietary habits of women during and before pregnancy should be considered when investigating the association between fetal undernutrition and the postnatal development of the child. Thus, our results are important from a clinical perspective and with regard to public health. Moreover, we believe that good maternal lifestyle habits during and before pregnancy contribute significantly to the prevention of childhood obesity.
